# The effect of exercise-induced muscle fatigue on gait parameters among older adults: a systematic review and meta-analysis

**DOI:** 10.1186/s11556-025-00370-1

**Published:** 2025-04-01

**Authors:** Paul Benjamin Voorn, Remco Oomen, Jacek Buczny, Daniël Bossen, Bart Visser, Mirjam Pijnappels

**Affiliations:** 1 Faculty of Health, Sport and Physical Activity, Centre of Expertise Urban Vitality, Amsterdam University of Applied Sciences, Amsterdam, Netherlands; 2 Department of Human Movement Sciences, Faculty of Behavioural and Movement Sciences, Amsterdam Movement Sciences Research Institute, Vrije Universiteit Amsterdam, Amsterdam, Netherlands; 3 Department of Experimental and Applied Psychology, Faculty of Behavioural and Movement Sciences, Vrije Universiteit Amsterdam, Amsterdam, Netherlands

**Keywords:** Exercise-induced fatigue, Muscle fatigue, Older adults, Gait, Meta-analysis

## Abstract

**Background:**

Exercise-induced fatigue is a common consequence of physical activities. Particularly in older adults, it can affect gait performance. Due to a wide variety in fatiguing protocols and gait parameters used in experimental settings, pooled effects are not yet clear. Furthermore, specific elements of fatiguing protocols (i.e., intensity, duration, and type of activity) might lead to different changes in gait parameters. We aimed to systematically quantify to what extent exercise-induced fatigue alters gait in community-dwelling older adults, and whether specific elements of fatiguing protocols could be identified.

**Methods:**

This systematic review and meta-analysis was conducted in accordance with the PRISMA guidelines. In April 2023, PubMed, Web of Science, Scopus, Cochrane and CINAHL databases were searched. Two independent researchers screened and assessed articles using ASReview, Rayyan, and ROBINS-I. The extracted data related to spatio-temporal, stability, and variability gait parameters of healthy older adults (55 +) before and after a fatiguing protocol or prolonged physical exercise. Random-effects meta-analyses were performed on both absolute and non-absolute effect sizes in RStudio. Moderator analyses were performed on six clusters of gait parameters (Dynamic Balance, Lower Limb Kinematics, Regularity, Spatio-temporal Parameters, Symmetry, Velocity).

**Results:**

We included 573 effect sizes on gait parameters from 31 studies. The included studies reflected a total population of 761 older adults (57% female), with a mean age of 71 (SD 3) years. Meta-analysis indicated that exercise-induced fatigue affected gait with a standardized mean change of 0.31 (*p* < .001). Further analyses showed no statistical differences between the different clusters, and within clusters, the effects were non-uniform, resulting in an (indistinguishable from) zero overall effect within all clusters. Elements of fatiguing protocols like duration, (perceived) intensity, or type of activity did not moderate effects.

**Discussion:**

Due to the (mainly) low GRADE certainty ratings as a result of the heterogeneity between studies, and possible different strategies to cope with fatigue between participants, the only conclusion that can be drawn is that older adults, therapist, and researchers should be aware of the small to moderate changes in gait parameters as a result of exercise-induced fatigue.

## Introduction

Muscle fatigue, or performance fatigability, is a natural consequence of physical activities and exercise (e.g., walking) and may lead to a reduction in physical performance [1], particularly in the older population [2]. Multiple reviews have been performed on the effects of exercise-induced (muscle) fatigue in older adults with regards to gait, functional tasks, dual task performance and standing balance. In general, these reviews show that exercise-induced fatigue negatively affects physical performance outcomes. For instance, gait stability-, gait variability-, standing balance, and spatio-temporal parameters change when fatigued [3–7], indicating that fatigue could lead to an increased risk of tripping and falls [8, 9]. However, the heterogeneity in fatiguing protocols and gait performance outcomes makes it hard to draw consistent conclusions on the consequences of exercise-induced fatigue on gait performance. As no meta-analysis has yet been conducted on the effects of exercise-induced fatigue on gait parameters in older adults, the pooled effects of gait parameters and their directions are currently unknown. Furthermore, in this study we aimed to evaluate the heterogeneity in fatiguing protocols between studies to pinpoint specific elements of fatiguing exercises that influence gait performance.

Generally, the heterogeneity stemming from fatiguing protocols accounts for the duration of the exercise, the (perceived) intensity of the exercise and/or the type of activity. For example, most fatiguing protocols either involve high intensity for short duration exercises or low intensity for long duration exercises [10–15]. It is suggested that these two combinations induce a different type of fatigue, namely peripheral fatigue or central fatigue, respectively [16–19], with different underlying pathways [20, 21]. The differences between fatiguing protocols is also found in the type of activity, which can be indicated by the number of muscles involved or the type of contraction(s). In previous reviews, a range from single muscle (isokinetic) exercises to more whole body, cyclic, activities such as walking are described [3–7]. Since muscle fatigue is specific to task demands, the type of activity, together with intensity and duration, are considered important factors for the limiting adjustments that come with fatigue [22–24].

This review aimed to make clear if, regardless of fatiguing protocol, exercise-induced muscle fatigue in general will change gait parameters in older adults. Furthermore, the variation in fatiguing protocols across studies could shed light on the question of whether elements of these protocols contribute to changes in specific gait parameters. It seems plausible that, depending on the type of activity, as well as the intensity or the duration of the exercise, exercise-induced fatigue leads to different outcomes of gait parameters. Therefore, the aim of our systematic review and meta-analysis was to evaluate and quantify to what extent exercise-induced muscle fatigue alters gait parameters in community-dwelling older adults and whether specific elements of fatiguing protocols (i.e., type of activity as well as intensity or duration of the exercise) lead to different outcomes. First, and in line with previous research, we expected that gait stability, gait variability, and spatio-temporal parameters change as a consequence of the fatiguing exercises, regardless of the elements of fatiguing protocols. Second, we hypothesized that when the intensity of the protocols was (perceived as) high, a more severe level of fatigue would result in significantly greater changes in gait parameters, compared to low or medium intensity protocols. Third, we expected to find greater changes in gait parameters after protocols with longer durations, since in general longer duration leads to more depletion and thereby more fatigue. Fourth, we hypothesized that protocols that used walking activities lead to greater changes in the gait parameters compared to non-walking protocols, due to task-specificity. Analyzing which elements of fatiguing exercises contribute to changes in gait could provide insight into how older adults should prevent themselves from adverse fatiguing effects. These findings might be of use for (preventive) interventions for falls in the older population.

## Methods

This systematic review and meta-analysis were set up following the Preferred Reporting Items for Systematic Reviews and Meta-analyses (PRISMA) 2020 guidelines [25], the Cochrane Handbook for systematic reviews of interventions [26] and the Open Science Guidelines [27]. It was pre-registered with the International Prospective Register of systematic reviews (PROSPERO) (registration number: CRD42022357662).

## Search strategy

In April 2023, a systematic literature search in PubMed, Web of Science, Scopus, Cochrane, and the Cumulative Index to Nursing and Allied Health Literature (CINAHL) was performed. The search strategy included a combination of medical subject headings (MeSH) terms and similar keywords concerning (1) the population: “old*”, “senior*”, “elder*”, (2) the intervention: “fatigue”, “muscle fatig*”, “peripheral fatig*”, (3) the outcomes: “walking”, “gait stability”, “step length” and (4) exclusion criteria: “Parkinson*”, “COVID*”, “Stroke”. The search strings were built up with the use of Boolean operators (“AND”, “OR”, and “NOT”) and adapted to specific search engines. The full search strategies for the different databases are found in the Supplementary Materials (1. Search plans). Although the Cochrane Handbook states that the “NOT” operator should be avoided [26], we did include the operator in this search and used the “NOT” operator in combination with the field code “Title” to filter out articles based on exclusion criteria in the title. Thereby, we aimed to remove a substantial number of irrelevant papers since many studies concerning muscle fatigue and gait parameters were conducted in populations with neurological and/or orthopaedical diseases and, hence, not relevant to our study’s aims. Additional filters or restrictions were not used. Next to the searches, all available reference lists were examined. Furthermore, a grey literature search by contacting authors from the field asking for unpublished data or ongoing data using standardized templates was performed, following the recommendations by Moreau and Gamble [27].

## Study selection

We included experimental, quasi-experimental, and observational studies for further screening when they met the following inclusion criteria: (1) participants were non- or pre-frail community-dwelling older adults (55 + years of age) without comorbidities that would influence walking abilities, such as cardiovascular-, orthopedic- or neurological diseases, and (2) effects of exercise-induced fatigue or prolonged activity on gait parameters were compared with a non-fatigued state within participants.

The results of the search were imported into EndNote 20 [28]. After removing duplicates, all eligible articles were imported into the algorithm-aided open science software ASReview Lab [29]. For the learning phase of the selection process, we utilized input from a scoping search and reviews from other authors in the same field. After reading the full texts of 20 articles, we marked 10 articles as relevant and 10 articles (near misses) as irrelevant. Two authors (PV and RO) independently screened titles and abstracts using ASReview Lab. Since the software re-orders the articles after every decision and moves relevant articles up in ranking, only a fragment of the full set of articles needed to be screened. We decided to screen at least 10% and then screen up to 100 consecutive irrelevant articles [30, 31], applying the default learning model: “Naïve Bayes, TF-IDF, Max” [32]. The articles identified as potentially eligible after screening of titles and abstracts, were full-text analyzed independently by two authors (PV and RO) using the open science software Rayyan [33].

During the screening and full-text analysis, discrepancies were discussed by the two authors and when necessary, a third author from the team was asked for help (BV). Interrater reliability was calculated for both title and abstract screening and full-text analysis after the first search (0.66 and 0.60, respectively). The R script, calculations, and CIs of the interrater reliability are found in the Supplementary Materials (2. Interrater-reliability (irr) screening, 2. Interrater-reliability (irr) fulltext, 2. irr_screening, and 2. irr_fulltext).

## Quality assessment

The articles that remained after the full-text analysis were assessed on risk of bias using the ROBINS-I tool [34, 35]. The risk of bias assessment and GRADE criteria were used to rate the quality of the studies found, from “very low” to “high” GRADE certainty ratings [36]. GRADE considerations include limitations of studies, inconsistencies, lack of precision, indirectness, and publication bias. Two authors (PV and RO) independently assessed the risk of bias, GRADE was done by one researcher (PV) and checked by another (RO). Differences were solved by discussion and when necessary, a third author (JB) was asked for help.

## Data extraction

From the eligible articles, two authors (PV and RO) extracted information about study design, participant demographics (average age and percentage of females), elements of fatiguing activity and protocol (i.e., duration, type of activity, (perceived) intensity), gait parameters (i.e., spatio-temporal-, gait variability-, gait stability- or joint kinematic-parameters), pre- and post-fatigue and their standard deviations. The data were saved in a coding form that was built in Microsoft Excel. The two authors (PV and RO) checked each other’s work and added or adjusted data when necessary. When possible, the extracted outcomes were converted to the same unit of measure (e.g., centimeters converted to meters). Not every paper included the required data. If attempts to obtain data from the authors failed, data was imputed or hand-measured. Hand-measuring was done if figures were available. The imputation of data was based on averaged values per variable across available studies and implemented only if values were missing at random; otherwise, incomplete study data were deleted case-wise.

## Data synthesis

To test the first hypothesis that gait parameters change in response to fatiguing protocols, first, the negative effect sizes were transformed into their absolute values, and second, the transformed negative effect sizes and positive effect sizes were pooled.

**Fig. 1:**
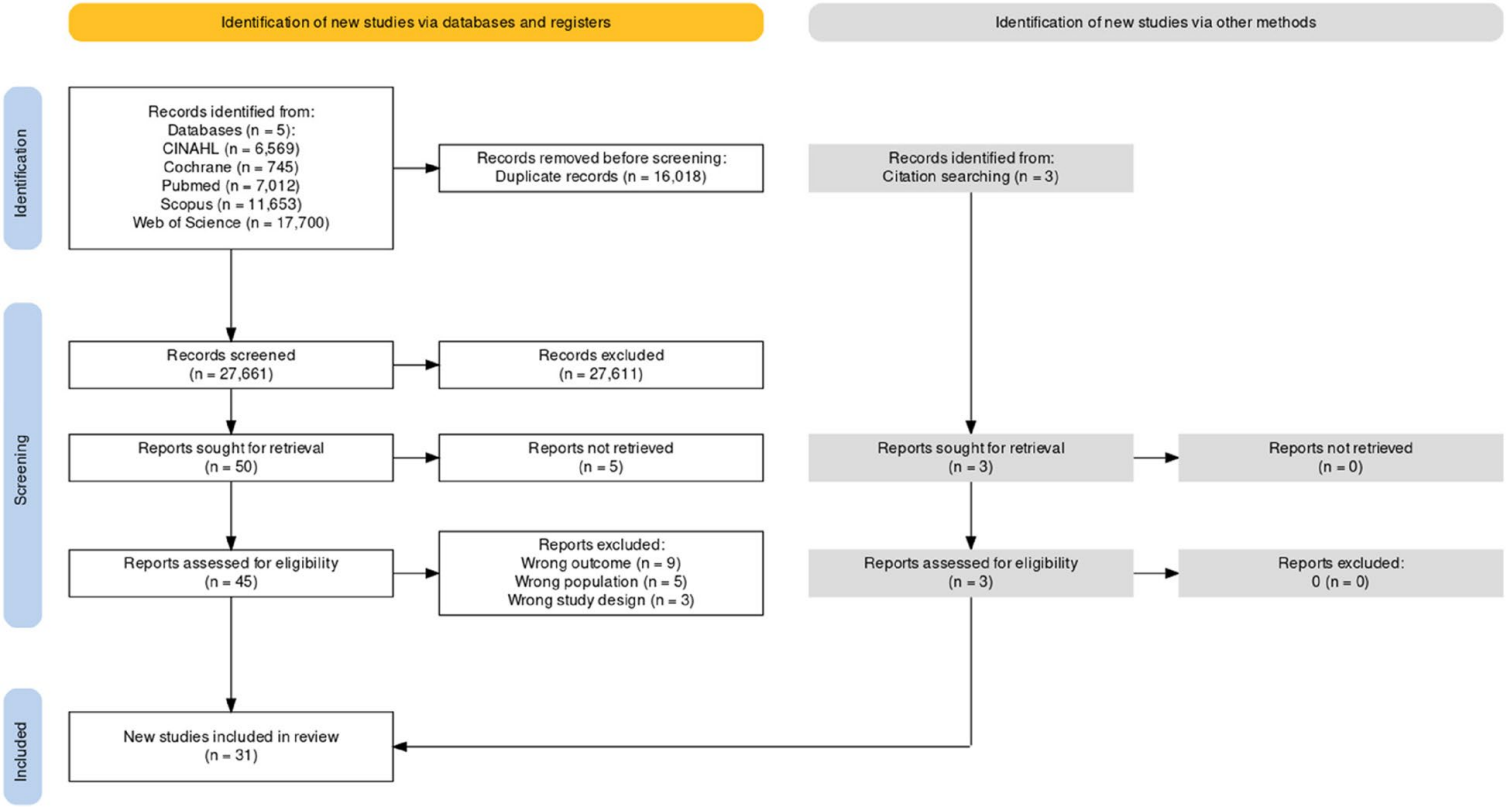
PRISMA flowchart showing how the 31 studies were included in this meta-analysis, figure created with PRISMA2020 [58]

To test the other hypotheses on the large number of effect-sizes, the non-absolute (i.e., raw) effect sizes were analyzed in clusters of gait parameters. All gait parameters were grouped into six clusters. These clusters were made based upon the previous research by Lindemann [37] and Dapp et al. [38] (Fig. 1 (p. 2)). In our classification: (1) gait parameters were placed into the Symmetry cluster when they matched with Lindemann’s paper or when they could be labelled in the Phase cluster as defined by Hollman [39]. Since the Phase cluster overlaps with Lindemann’s Symmetry cluster [38] all data was merged into the same cluster. (2) Gait-stability outcomes were grouped into the Dynamic Balance cluster. (3) Furthermore, the Foot Movement cluster was renamed to Lower Limb Kinematic cluster, and combined foot movement parameters with other lower limb kinematic parameters. For clarity purposes, (5) the Walking Capacity cluster and (6) the Coordination cluster were renamed to Velocity cluster and Spatio-temporal Parameters cluster respectively.

Only if ten or more effect sizes from different studies were available in the same cluster, results were pooled for meta-analysis.

### Statistical analysis

#### A priori analysis

Our a priori power analysis showed that at least 47 studies were needed (see the Supplementary Materials (6. Power analysis a priori) for more details). Regardless of the number of articles, the intended subgroup- (i.e., per cluster) and moderator analysis, via meta-regression, was performed.

#### Main analysis

It was expected that the included studies would vary in the methods used to induce fatigue, as well as in the measurements and type of gait parameters. Because of this, the analysis was conducted using the standardized raw mean change (SMCR) as the effect size measure and random-effects model was used to fit to the data. If between-measurement correlation related to a given SMCR was not reported, the correlation was imputed from an average correlation of the available data. In case of a substantial amount of missing correlation data, we rerun analysis with different imputed between measurements correlations (i.e., *r* = 0.00, *r* = 0.25, *r* = 0.50, *r* = 0.75, and *r* = 0.90), assuming that the true SMCR depends strongly on the value of the correlations between measurements (cf. [40]).

The level of heterogeneity (i.e., τ^2^) was estimated using the restricted maximum-likelihood estimator (REML) [41]. In addition to the estimate of τ^2^, the *Q*-test for heterogeneity [42], the *I*^*2*^ statistic [43] and prediction intervals were reported. The analysis was carried out using open science software R [44], RStudio (version 4.2.3) [45], and the *metafor* R package (version 4.4–0) [46]. Additional R packages that were used are: *clubSandwich* [47]*, dmetar* [48]*, tidyverse* [49] and *gridExtra* [50]. A multilevel model was applied to account for the dependencies between effect sizes reported in the same study.

#### Moderator analysis

Per cluster, moderator analyses were done to test if the duration of the protocols (longer than 10 min vs. shorter than 10 min), the rate of (perceived) exertion by the participants ((low vs. moderate vs. high), based on the RPE scale (low = lowest 33% of RPE scale, moderate = middle 33% of RPE scale, high = highest 33% of RPE scale), on heart rate, decrease in muscle capacity, on the description of the fatiguing protocol with words like until exhaustion, or a combination of these factors), and type of activity (walking vs. non-walking) affected the changes in gait parameters. For visualization of the findings, the *orchaRd* R package [51] was used to create orchard plots and *metafor* [46] to create forest plots.

#### Sensitivity analysis

To test the level of publication bias, for example, *p*-hacking analysis, the following R packages were used: *metaplus* [52], *publicationbias* [53], *phacking* [54, 55] and *multibiasmeta* [56].

#### Post hoc power analysis

A post hoc power analysis was conducted using the *POMADE* R package [57]. When the project started, power analysis for multilevel models was not available, so we decided to run a power analysis once *POMADE* became available. This additional power analysis is a more precise estimation of power than the initial power analysis conducted before the current project started, as the *POMADE* procedure specifically accounted for dependencies between the effect size.

## Results

### Study selection

The search resulted in 43,679 studies. Duplicates were removed, and 27,661 articles were screened using ASReview. After screening 10% of the found articles, we already had around 1500 consecutive irrelevant articles, leading to the decision to stop. The screening resulted in 50 articles that were manually labeled as relevant, and 2767 articles were manually labeled as irrelevant. All other articles were labeled irrelevant by the machine learning algorithm. After screening of full-text, 28 articles were included. Reasons for exclusion were wrong study population, inappropriate study design, irrelevant outcomes, or incomplete data. The citation search resulted in one additional study and e-mail contact with authors yielded two additional articles. Eventually, we included 31 articles (Fig. 1).

## Quality Assessment

**Fig. 2:**
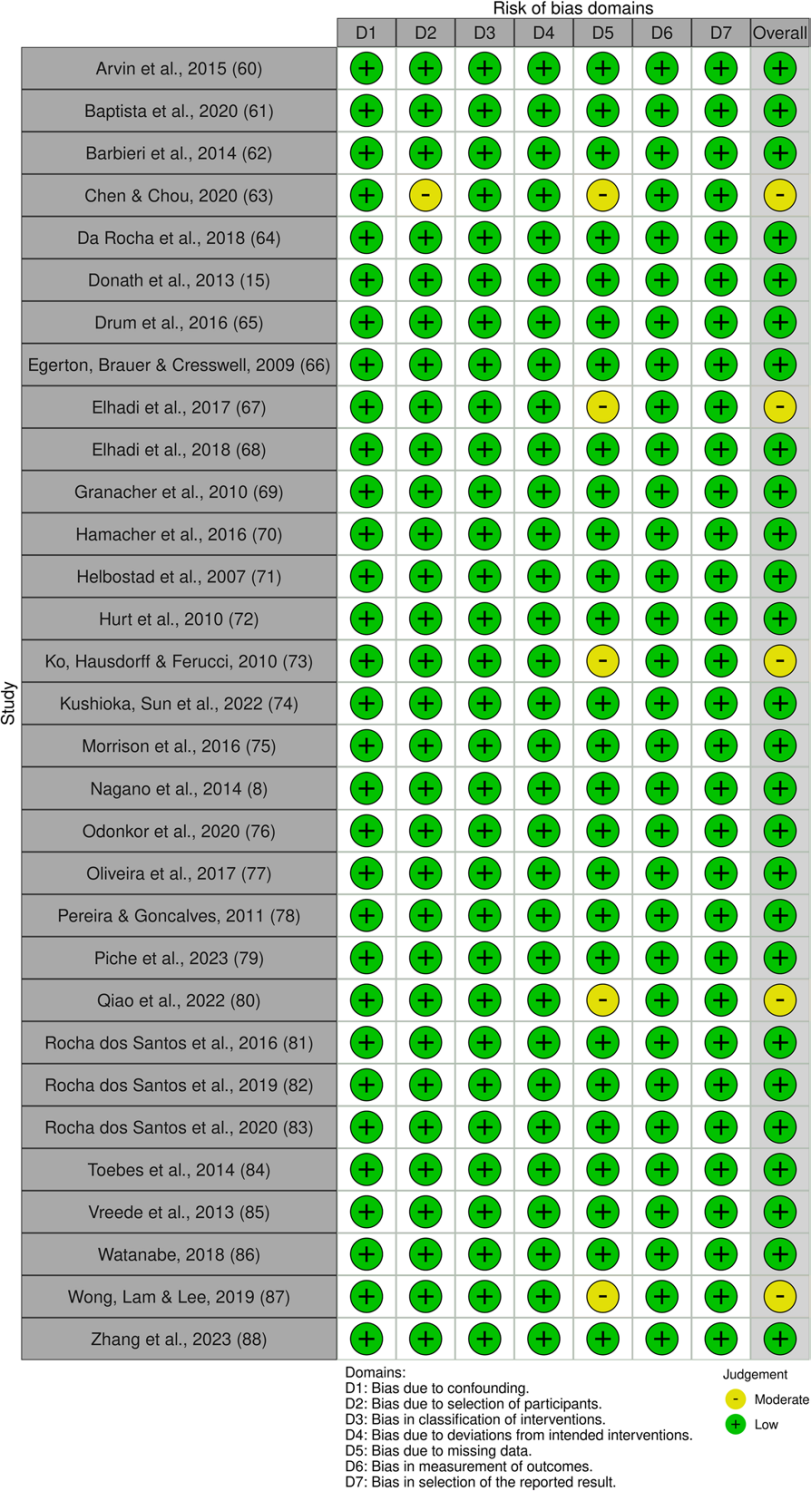
Risk of bias assessed with the ROBINS-I tool, figure created with robvis [59]

Five out of 31 studies had a moderate risk of bias rating, all other studies were considered to have a low risk of bias (see Fig. 2). The GRADE certainty ratings indicated low risk for all outcomes combined. In the Dynamic Balance and Spatio-temporal Parameters cluster the GRADE certainty rating was considered “very low”. The ratings were considered “low” in the Lower Limb Kinematics, Regularity, and Symmetry clusters and the GRADE rating turned out to be “moderate” in the Velocity cluster. More details on GRADE can be found in the Supplementary Materials (3. GRADE outcomes table).

## Study characteristics

A total of 573 effect sizes on gait parameters were included, with an average of 18.5 effect sizes per study. The included studies were conducted on a total population of 761 older adults with a mean age of 70.9 (± 2.8) years of age, and 57% of all participants were female. On average, there were 24.5 participants per study. Study characteristics are in the summary of findings table (Table 1; and for more details view the Supplementary Materials (4. Summary of Findings table)).

**Table 1:** Summary of findings table. Alphabetically ordered

Study details			Participants			Fatiguing exercise			Outcomes		
**Author, year**	**Study design**	**Overall Risk of bias**	**N (group label)**	**Sex (% female)**	**Age (years)**	**Fatiguing task**	**Duration (min)**	**(Perceived) intensity**	**Walking task**	**Cluster**	**#Effect Sizes per cluster (of which significant)**
Arvin et al.,, 2015 [60]	pre-post	Low	17	71	73,2	Hip—Abduction (unilateral)	2,94	> 8 a	Treadmill	DB	6
										Reg	3 (2)
										ST	1
Baptista et al., 2020 [61]	pre-post	Low	33	100	70,5	Stair climbing and descending	NI	NI	Overground at 6-m long walkway	ST	4
										Vel	2
Barbieri et al., 2014 [62]	pre-post	Low	20 (G60)	0	64	Bilateral sit-to-stand	4,8	18.6 b	Overground (barefoot) at 8-m long walkway	DB	1 (1)
										ST	2 (2)
										Vel	1 (1)
			20 (G700)	0	74,5	Bilateral sit-to-stand	3	17.4 b	Overground (barefoot) at 8-m long walkway	DB	1 (1)
										ST	2 (2)
										Vel	1 (1)
Chen & Chou, 2022 [63]	pre-post	Moderate	17	52,9	69,5	Bilateral sit-to-stand	17,36	17.6 b	Overground walking for 2 min at an oval with 15-m straight walkway	DB	3
										Vel	1
Da Rocha et al., 2018 [64]	pre-post	Low	15 (active)	80	67	Walking: self selected speed	30	NI	Treadmill	DB	1
										ST	6 (1)
			15 (sedentary)	100	68	Walking: self selected speed	30	NI	Treadmill	DB	1
										ST	6 (1)
Donath et al., 2013 [15]	pre-post	Low	19 (day 1)	52,6	64,6	Walking: maximal exhaustive ramp like exercise on a treadmill	22,8	9.5 a	Overground 3 times at 12-m long walkway	DB	1
										Reg	2
										ST	3
										Sym	1
			19 (day 2 or 3)	52,6	64,6	Walking: submaximal 2-km exercise test	27,7	4a	Overground 3 times at 12-m long walkway	DB	1
										Reg	2
										ST	3
										Sym	1
Drum et al., 2016 [65]	Prolonged activity	Low	36	44,4	62	Walking: treadmill (incline) walk	40	2.1 a	Treadmill	Reg	2
										ST	3
										Sym	1
Egerton, Brauer & Cresswell, 2009 [66]	pre-post	Low	10 (balance impaired)	80	81,6	Functional mobility tasks	14	3.46 a (leg muscle only)	Overground at 8-m long walkway	Reg	2
										ST	2 (1)
										Sym	1
										Vel	1
			10 (Healthy old)	60	71,2	Functional mobility tasks	14	1.89 a (leg muscle only)	Overground at 8-m long walkway	Reg	2
										ST	2
										Sym	1
										Vel	1
Elhadi et al., 2017 [67]	pre-post	Moderate	14 (A)	35,6	69,5	Walking: self selected speed	30	1.1 a	Overground at 8-m long walkway	LLK	46
										ST	7
										Vel	1
							60	1.4 a	Overground at 8-m long walkway	LLK	46 (4)
										ST	7
										Vel	1
			10 (B)	30	70	Walking: self selected speed	30	3.6 a	Overground at 8-m long walkway	LLK	46 (4)
										ST	7 (5)
										Vel	1
							60	4.2 a	Overground at 8-m long walkway	LLK	46 (11)
										ST	7 (6)
										Vel	1
Elhadi et al., 2018 [68]	pre-post	Low	15	21,4	71,6	Walking: self selected speed	30	2.5 a	Overground at 8-m long walkway	LLK	30 (8)
										ST	7
										Vel	1
							60	3.4 a	Overground at 8-m long walkway	LLK	30 (11)
										ST	7 (2)
										Vel	1
Granacher et al., 2010 [69]	pre-post	Low	16	50	71,9	Isokinetic knee flexion and extension	NI	16.1 b	Overground at 10-m long walkway	Reg	1 (1)
										ST	1
										Vel	1
Hamacher et al., 2016 [70]	pre-post	Low	18	NI	69	Cycle ergometer submaximal task	NI	until submaximal exhaustion (18 b)	Treadmill	DB	1 (1)
Helbostad et al., 2007 [71]	pre-post	Low	22	77,2	78,2	Bilateral sit-to-stand	Between 5 and 15 min	Until exhaustion	Overground at 7-m long walkway	DB	5 (2)
										Reg	4 (2)
										ST	1
										Vel	1
Hurt et al., 2010 [72]	pre-post	Low	11	63,6	60,6	Walking: self selected speed	10	NI	Treadmill	DB	4
Ko, Hausdorff & Ferucci, 2010 [73]	pre-post	Moderate	183	48	73	Walking: different trails	30	NI	Overground at 10-m long walkway	DB	1
										ST	2
										Sym	1
										Vel	1
Kushioka, Sun et al., 2022 [74]	Prolonged activity	Low	7	50	65,9	Walking: 6-min walking test	6	NI	Overground at 20-m long walkway	Reg	12
										ST	6
										Sym	5
										Vel	1
Morrison et al., 2016 [75]	pre-post	Low	15 (group 60–69)	53,3	64,2	Walking: Treadmill walking	3 × 5 min	16.4 b	Overground at 20-ft long walkway	ST	1 (1)
										Vel	1 (1)
			15 (Group 70–79)	53,3	74,5	Walking: Treadmill walking	3 × 5 min	15.36 b	Overground at 20-ft long walkway	ST	1 (1)
										Vel	1 (1)
Nagano et al., 2014 [8]	pre-post	Low	11	NI	74,2	Walking: self- selected maximum speed	6	12.1b (average during activity)	Treadmill	DB	2
										LLK	2 (2)
										ST	2
										Sym	2
Odonkor et al., 2020 [76]	Prolonged activity	Low	5	50	67,8	Walking: self selected speed	10	NI	Overground	Reg	12
										ST	6
										Sym	5
										Vel	1
Oliveira et al., 2017 [77]	pre-post	Low	23	NI	71,1	Walking: Fast walking	20	NI	Treadmill	DB	2 (1)
										LLK	6 (2)
										Reg	8
										ST	3 (2)
Pereira & Gonçalves, 2011 [78]	Prolonged activity	Low	8	100	72,6	Walking: self selected speed	20	NI	Treadmill	ST	2 (2)
Piche et al., 2023 [79]	pre-post	Low	20	NI	75,8	Bilateral sit-to-stand	NI	NI	Overground at 10-m long walkway	Reg	4
										ST	6
										Sym	3
										Vel	1
Qiao et al., 2022 [80]	Prolonged activity	Moderate	59 (day 1 Fast paced)	55,6	78,3	Walking: 400 m fast-paced walking	5,49	NI	Overground at 20-m long walkway	ST	1
			56 (day 2 Normal paced)	55,6	78,3	Walking: 400 m self-selected speed	6,35	NI	Overground at 20-m long walkway	ST	1
Rocha dos Santos et al., 2016 [81]	pre-post	Low	10 (Active)	NI	67,5	Bilateral sit-to-stand	6,81	17.5 b	Overground at 8-m long walkway	DB	1
										ST	2
										Sym	1
										Vel	1
			10 (Inactive)	NI	71,4	Bilateral sit-to-stand	2,04	19.2 b	Overground at 8-m long walkway	DB	1
										ST	2
										Sym	1
										Vel	1
Rocha dos Santos et al., 2019 [82]	pre-post	Low	12	40	71	Bilateral sit-to-stand	4,47	19.58 b	Treadmill	DB	7 (1)
										Reg	3 (1)
										ST	4 (3)
Rocha dos Santos et al., 2020 [83]	pre-post	Low	12	41,7	71	Bilateral sit-to-stand	4,47	18 b	Treadmill	DB	1
										ST	4
Toebes et al., 2014 [84]	pre-post	Low	10	60	63,4	unilateral knee bending	NI	Until exhaustion	Treadmill	DB	5
										Reg	3
										ST	6
										Sym	1
Vreede et al., 2013 [85]	pre-post	Low	11	16,7	68	Walking: 6-min walking test	6	9.2 b	Overground at 10-m long walkway	LLK	6 (3)
										ST	2
										Vel	1 (1)
Watanabe, 2018 [86]	Prolonged activity	Low	6 (Old with falling)	0	69	Walking: self selected speed	20	NI	Treadmill	LLK	1
										ST	2
										Sym	2
			7 (Old without falling)	0	73,3	Walking: self selected speed	20	NI	Treadmill	LLK	1 (1)
										ST	2
										Sym	2
Wong, Lam & Lee, 2020 [87]	Prolonged activity	Moderate	16	31,2	70	Walking: self selected speed	30	NI	Overground at 10-m long walkway	ST	3
										Sym	4 (1)
										Vel	1
							60	NI	Overground at 10-m long walkway	ST	3
										Sym	4 (1)
										Vel	1
Zhang et al., 2023 [88]	Prolonged activity	Low	18	50	60,4	Walking: Brisk walking	60	6.78 a	Treadmill	LLK	1
										ST	2 (2)
										Sym	4

a = (Perceived) intensity measured by CR-10, b = (Perceived) intensity measured by CR-20, *DB* Dynamic Balance, *ES* Effect sizes, *LLK* Lower Limb Kinematics, *NI* no information, *Reg* Regularity, *ST* Spatio-temporal Parameters, *Sym* Symmetry, *Vel* Veloci

Since both Odonkor et al. [76] and Kushioka et al. [74] reported on the same dataset and two papers of Rocha dos Santos et al. [82, 83] also used the same dataset, the outcomes were treated in our analysis as if they were from one study. Therefore, the analysis was conducted on 29 datasets instead of 31 studies.

### Meta-analysis outcomes

Since many between-measurement correlations could not be retrieved, the analyses were rerun using different imputed between-measurement correlations, namely *r* = 0.00, *r* = 0.25, *r* = 0.50, *r* = 0.75, and *r* = 0.90. The focus was placed on results which were based on a conservative assumption of the between-measurement correlations; we only report here the SMCR calculated based on *r* = 0.25. The analyses with the different various between-measurement correlations (*r* = 0.00, *r* = 0.50, *r* = 0.75, and *r* = 0.90) can be found in the Supplementary Materials (7. Outcomes meta-analysis).

Imputation of data was done for 9 effect sizes; only standard deviations and no mean effects were imputed.

#### Overall effects on gait parameters

**Fig. 3:**
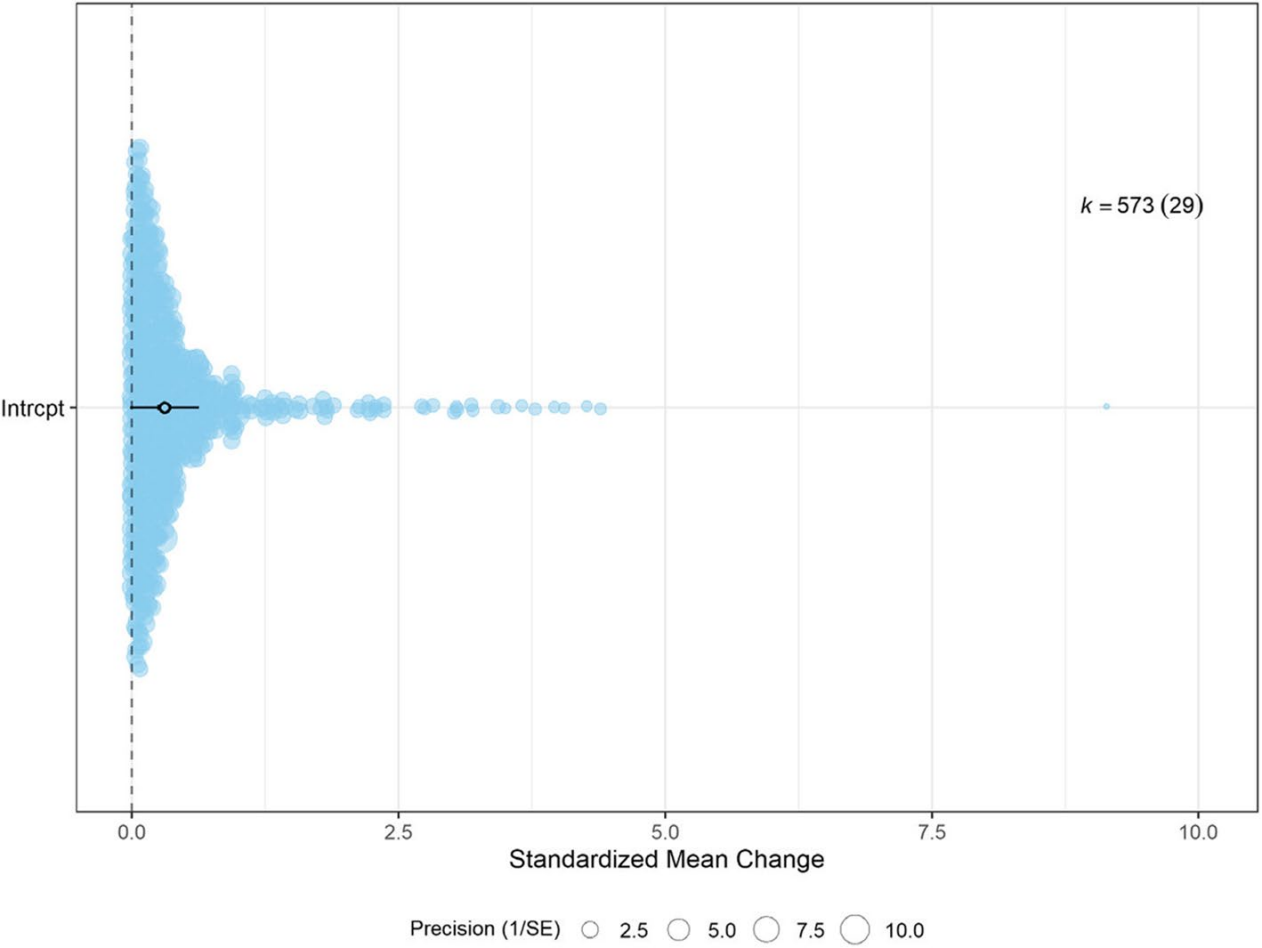
Orchard plot of the absolute data with all 573 gait parameters, data from 29 data sets. The orchard shows the estimate as an open circle and the confidence interval as the horizontal black line. The number of effect sizes is represented by k and the number of data sets is between parentheses. The size of the color filled circles represents precision of the effect size

When analyzing absolute values of gait parameters, a minimal to moderate change (SMCR = 0.31, 95% confidence interval (CI) [0.24, 0.37], *p* < 0.01*, k* = 573) was detected when post-fatiguing walking was compared to pre-fatiguing walking (Table 2 and Fig. 3).

**Table 2:** Changes in gait as result of exercise-induced fatigue, analysis on absolute data (*r* = 0.25)

**Absolute data**	Summary effect, 95% Confidence Interval (CI)					Tests of Heterogeneity														
Parameter *(k, n)*	Effect size SMCR (SE)	95% CI LB	95% CI UB	*t* *(df)*	*p*	95% PI LB	95% PI UB	τ^2^-between	τ^2^-within	*I*^*2*^-between	*I*^*2*^-between 95% CI LB	*I*^*2*^-between 95% CI UB	*I*^*2*^-within	*I*^*2*^-within 95% CI LB	*I*^*2*^-within 95% CI UB	*I*^*2*^-total	*I*^*2*^-total CI LB	*I*^*2*^-total CI UB	Q	*p*-val
All gait parameters (573, 29)	0.31 (0.03)	0.24	0.37	9.21 (572)	** < *.001***	−0.02	0.63	0.02	0.01	15.55	7.88	28.63	5.20	0.38	11.91	19.29	10.91	31.67	876.81	** < *.001***

*k* effect sizes, *n* datasets, *SMCR* Standardized mean change, *SE* standard error, *CI* confidence interval, *df* degrees of freedom, *PI* prediction interval, *LB* lower bound, *UB* upper bound

**Fig. 4:**
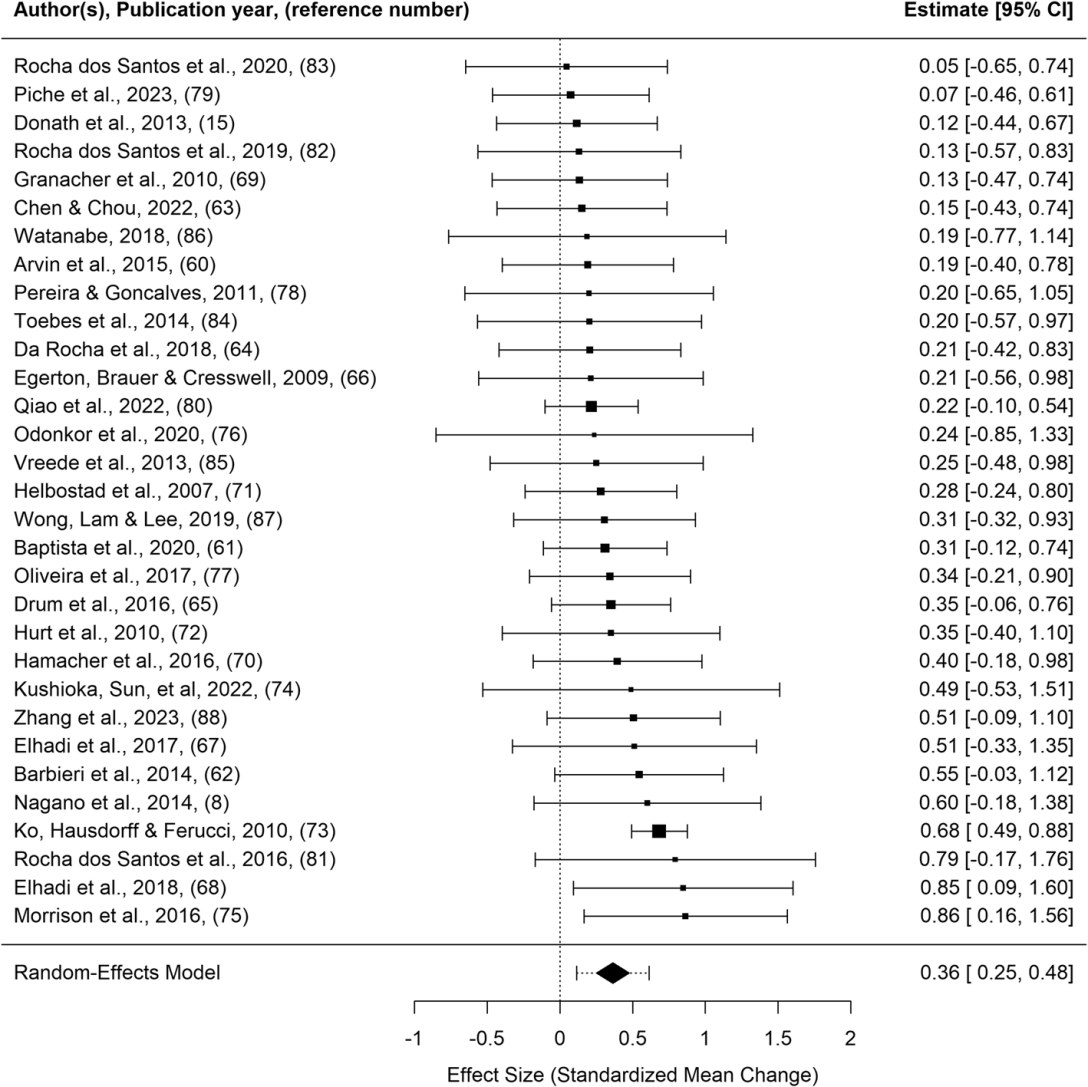
Forest plot with aggregated absolute outcomes per study, listed by effect size. Note that the estimate in this forest plot is different than the SMCR in Table 2; the outcomes in this forest plot were aggregated per study before analysis

For visualization purposes, a forest plot with absolute gait parameters aggregated per study is presented (Fig. 4).

#### Clustering effect sizes

All the gait parameters were grouped into six different clusters: Dynamic Balance, Lower Limb Kinematics, Regularity, Spatio-temporal Parameters, Symmetry, and Velocity. See Table 3 for an overview of the clusters, underlying gait parameters, and distribution of effect sizes.

**Table 3:** Overview of clusters of gait parameters

Dynamic balance* (45, 56%* +*)*	Lower Limb Kinematics *(261, 51%* +*)*	Regularity *(60, 45%* +*)*	Spatio-temporal Parameters *(139, 42%* +*)*	Symmetry *(42, 60%* +*)*	Velocity *(26, 65%* +*)*
• Step/Stride width *(19, 53%* +*)* • Step/Stride width variability *(6, 100%* +*)* • Gait stability outcomes *(20, 45%* +*)*: - Center of Mass displacement - Center of Mass peak velocity - Detrended Fluctuations Analysis (DFA) - Lyapunov exponent (LDE) - Multi-scale Sample Entropy - Peak trunk velocity - Trunk accelerations	• Minimal foot/toe clearance *(5, 0%* +*)* • Lower Limb Kinematics *(256, 52%* +*):* - Joint angle - Joint moment - Joint power	• Harmonic ratio A-P and M-L *(2, 0%* +*)* • Trunk repeatability *(3, 33%* +*)* • Variability (SD / CoV) *(55, 47%* +*)*: - Cadence - Cycle-time - Double support - Joint angle - Single support - Stance phase - Step/Stride length - Step/Stride time - Swing phase - Swing time - Velocity	• Cadence *(34, 32%* +*)* • Single support time (2, 50*%* +) • Stance time *(19, 26%* +*)* • Step/Stride length *(52, 58%* +*)* • Step/Stride time *(17, 47%* +*)* • Swing time *(15, 27%* +*)*	• Asymmetry Index (ASI) *(6, 83%* +*)* • Double support time *(1, 100%* +*)* • Phase outcomes (% of gait cycle) *(35, 54%* +*)*: - Double support phase - Gait-specific phase - Minimal foot clearance - Single support phase - Stance phase - Swing phase - Toe off	• Velocity *(26, 65%* +*)*

*Indicated between parentheses is the number of effect sizes and the percentage of positive effect sizes*

#### Effects on clustered data

Analysis of the clusters with absolute data showed that fatigue had the larger effect on Velocity (SMCR = 0.42, 95% CI [0.29, 0.54], *p* < 0.001, *k* = 26, *n* = 15)), and the least effect on Dynamic Balance (SMCR = 0.23, 95% CI [0.12, 0.34], *p* < 0.001, *k* = 45, *n* = 14). Nonetheless, moderator analysis showed no statistical differences between the clusters (*p* = 0.19; view Table 4 for more details).

**Table 4:** Moderator analysis of clustered absolute data (*r* = 0.25)

**Absolute data**	Summary effect and 95%CI					Test of moderation			
Moderator *(k, n)*	Effect size SMC (SE)	95% CI LB	95% CI UB	*t*	*p*	*F (df1, df2)*	*p*	*R*^*2*^	*I*^*2*^_within_ / *I*^*2*^_between_ / *I*^*2*^_total_
Clusters (*573, 29)*						1.50 (5, 567)	0.19	0.06	2.10 / 14.62 / 16.71
Dynamic balance *(45, 14)*	0.23 (0.06)	0.12	0.34	4.19	** < *.001***				
Lower Limb Kinematics *(261, 7)*	0.32 (0.05)	0.21	0.42	6.02	** < *.001***				
Regularity *(60, 11)*	0.30 (0.06)	0.19	0.41	5.29	** < *.001***				
Spatio-temporal Parameters *(139, 26)*	0.33 (0.04)	0.25	0.41	8.36	** < *.001***				
Symmetry *(42, 12)*	0.25 (0.06)	0.13	0.38	4.16	** < *.001***				
Velocity *(26, 15)*	0.42 (0.07)	0.29	0.54	6.35	** < *.001***				

*k* effect sizes, *n* datasets, *SMCR* Standardized mean change, *SE* standard error, *CI* confidence interval, *df* degrees of freedom, *PI* prediction interval, *LB* lower bound, *UB* upper bound

Analysis of non-absolute data by means of the random effects model analysis, showed that in all the clusters the pooled estimate indicated “no to a small change” effects (minimum SMCR = –0.01, 95% CI [–0.10, 0.09], *p* = 0.90, *k* = 136, *n* = 26 (Spatio-temporal Parameters); maximum SMCR = 0.24, 95% CI [–0.01, 0.49], *p* = 0.06, *k* = 26, *n* = 15 (Velocity)). Nevertheless, pre- vs. post-fatiguing changes in all the clusters were statistically non-significant and thereby the effects are considered non-distinguishable from zero (Table 5).

**Table 5:** Changes in gait as result of exercise-induced fatigue, per cluster of gait parameters with non-absolute data (*r* = 0.25)

**Non-absolute data**	Summary effect, 95% CI					Tests of Heterogeneity														
Cluster of gait parameters *(k, n)*	Effect size SMCR (SE)	95% CI LB	95% CI UB	*t* *(df)*	*p*	95% PI LB	95% PI UB	τ^2^-between	τ^2^-within	*I*^*2*^-between	*I*^*2*^-between 95% CI LB	*I*^*2*^-between 95% CI UB	*I*^*2*^-within	*I*^*2*^-within 95% CI LB	*I*^*2*^-within 95% CI UB	*I*^*2*^-total	*I*^*2*^-total 95% CI LB	*I*^*2*^-total 95% CI UB	Q	*p*-val
Dynamic Balance (45, 14)	0.03 (0.07)	−0.11	0.18	0.47 (44)	0.64	−0.39	0.46	0.04	0.00	31.52	5.75	61.50	0.00	0.00	23.60	31.52	9.37	61.50	48.87	0.28
Lower Limb Kinematics (261, 7)	−0.10 (0.09)	−0.27	0.07	−1.16 (260)	0.25	−1.13	0.94	0.02	0.25	10.74	0.05	56.82	65.97	57.37	73.03	67.31	58.77	77.07	743.51	** < *0.001***
Regularity (60, 11)	0.03 (0.06)	−0.09	0.15	0.47 (59)	0.64	−0.25	0.30	0.02	0.00	11.54	0.00	42.69	0.00	0.00	28.10	11.54	0.00	42.69	65.99	0.25
Spatio-temporal Parameters (139, 26)	−0.01 (0.05)	−0.10	0.09	−0.13 (138)	0.90	−0.70	0.69	0.01	0.11	11.03	0.00	46.02	53.43	27.82	67.97	55.98	40.32	68.50	411.13	** < *0.001***
Symmetry (42, 12)	−0.06 (0.07)	−0.20	0.08	−0.87 (41)	0.39	−0.59	0.47	0.00	0.06	0.00	0.00	63.83	38.74	0.00	65.52	38.74	–	–	76.80	** < *0.001***
Velocity (26, 15)	0.24 (0.12)	−0.01	0.49	2.00 (25)	0.06	−0.61	1.10	0.16	0.00	65.31	31.48	83.89	0.00	0.00	58.57	65.31	41.51	83.89	83.98	** < *0.001***

*k* effect sizes, *n* datasets, *SMCR* Standardized mean change, *SE* standard error, *CI* confidence interval, *df* degrees of freedom, *PI* prediction interval, *LB* lower bound, *UB* upper bound

**Fig. 5:**
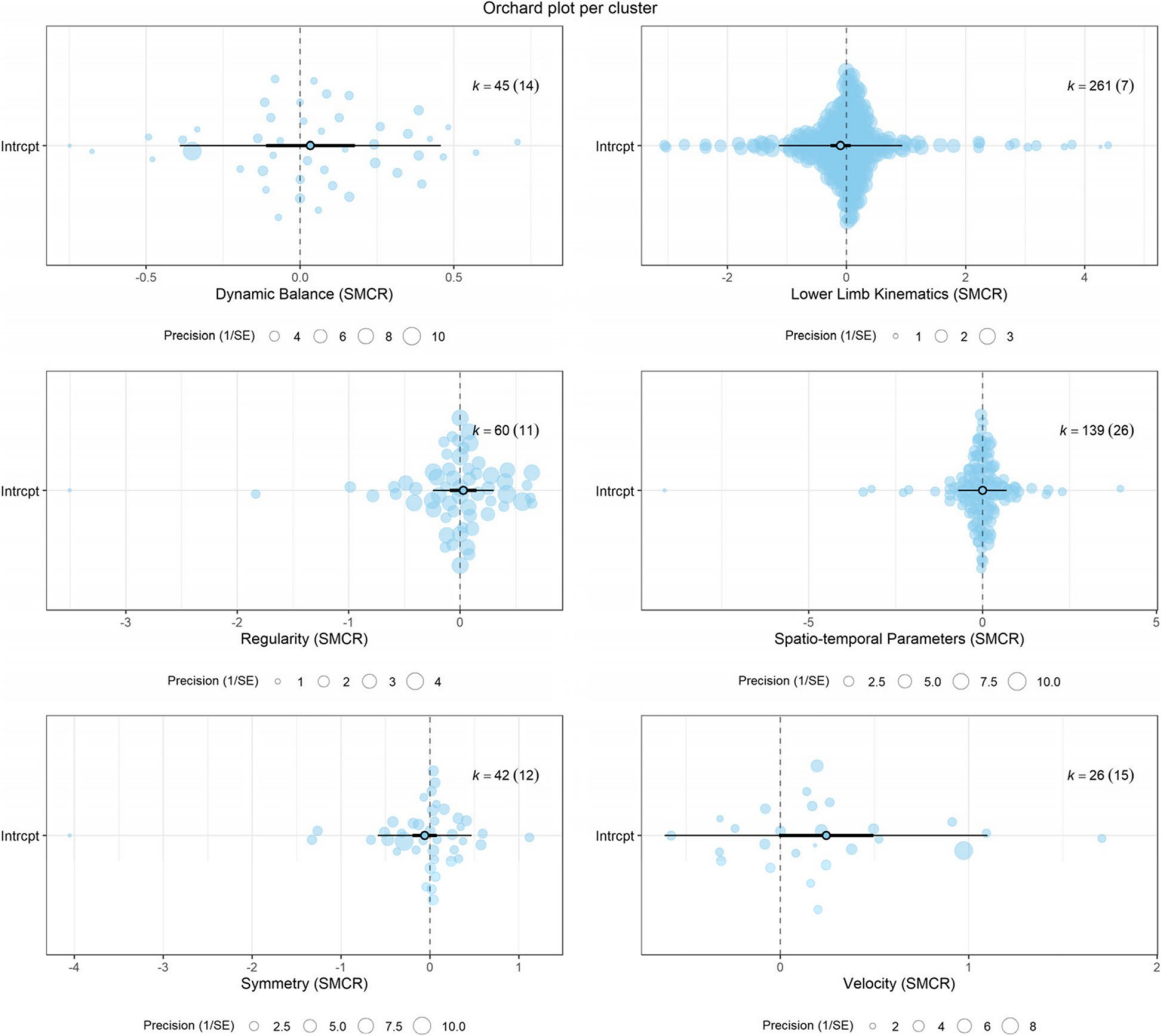
Orchard plots per cluster with non-absolute data, indicating that in all clusters the effects are considered non-distinguishable from zero and statistically non-significant. Besides, heterogeneity is assumed to be present in all clusters. This is reflected by the estimate (open circle), the confidence interval (horizontal thick black line), and the prediction interval (horizontal thin line). The number of effect sizes is represented by k and the number of data sets is between parentheses. The size of the color filled circles corresponds with the precision of the effect size

The variance in the observed effects that reflects variance in true effect can be derived from the values of* I*^*2*^ [89]*.* The proportion of variance is shown to be “might not be important” for the clusters: Regularity, Dynamic Balance, and Symmetry (approximately 12%, 32%, and 39%, respectively) and “may represent substantial heterogeneity” for Spatio-temporal Parameters, Velocity and Lower Limb Kinematics (approximately 56%, 65%, and 67%, respectively) [26]. Although *Q*-statistic was not significant in the Dynamic Balance and Regularity clusters, we did assume that heterogeneity was present in all the clusters. One of the reasons was the range in the effect sizes, as visualized in the orchard plots, and another reason was the values of the prediction intervals (PIs), which included opposing effects in all the clusters (Fig. 5).

##### 3.4.3.1 Moderator analysis

An analysis of the three moderators (i.e., type of activity, duration, and (perceived) intensity) on each of the six clusters resulted in only one statistically significant moderator (Tables 6, 7 and 8). Within the Regularity cluster (perceived) intensity turned out to be a significant moderator (*p* < 0.01, *k* = 24, *n* = 8). In particular, the type of protocol that was (perceived) as Low Intensity showed an increase in regularity outcomes post-fatiguing protocol/task (SMCR = 0.49, 95% CI [0.18, 0.80], *p* < 0.01, *k* = 2, *n* = 1) compared to general outcomes of the Regularity cluster (SMCR = 0.03, 95% CI [–0.09, 0.15], *p* = 0.64, *k* = 60, *n* = 11). However, the number of the effect sizes from these data sets was low, which might be too low to achieve sufficient statistical power for such an analysis (Table 8).

**Table 6:**
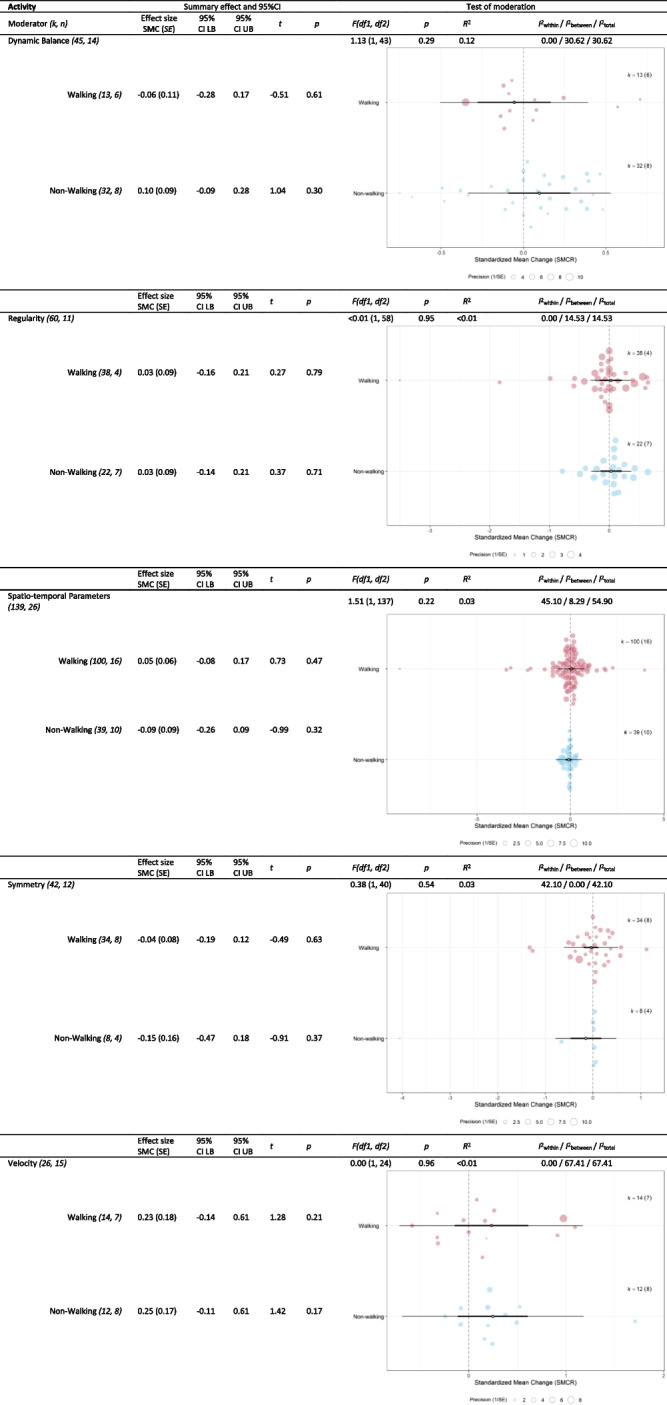
Outcomes of moderator analyses of ‘type of activity’ on different clusters of gait parameters (*r* = 0.25)

*CI* confidence interval, *df* degrees of freedom, *k* effect sizes, *LB* lower bound, *n* datasets, *PI* prediction interval, *SMCR* Standardized mean change, *SE* standard error, *UB* upper bound. In the orchard plots the estimate (open circle), the confidence interval (horizontal thick black line), and the prediction interval (horizontal thin line) can be distinguished. Furthermore, the size of the color filled circles corresponds with the precision of the effect size. Note that the Lower Limb Kinematics cluster is not in this table since all fatiguing protocols used the same type of activity

**Table 7:**
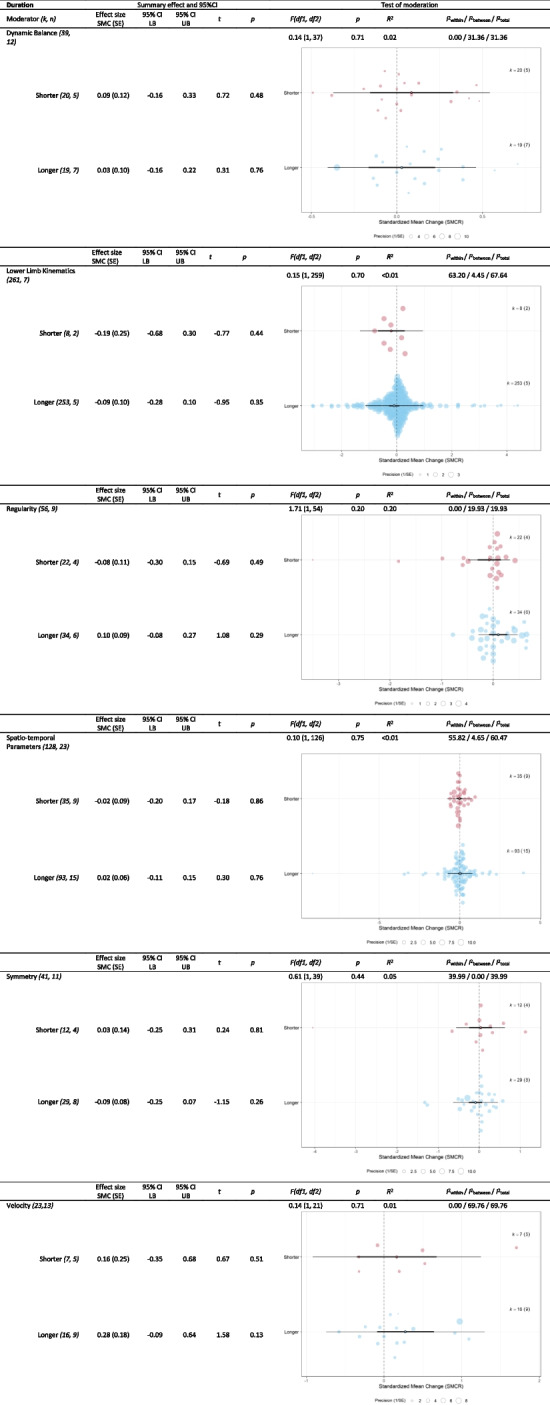
Outcomes of moderator analyses of ‘duration’ on different clusters of gait parameters (*r* = 0.25)

*CI* confidence interval, *df* degrees of freedom, *k* effect sizes, *LB* lower bound, *n* datasets, *PI* prediction interval, *SMCR* Standardized mean change, *SE* standard error, *UB* upper bound. In the orchard plots the estimate (open circle), the confidence interval (horizontal thick black line), and the prediction interval (horizontal thin line) can be distinguished. Furthermore, the size of the color filled circles corresponds with the precision of the effect size

**Table 8:**
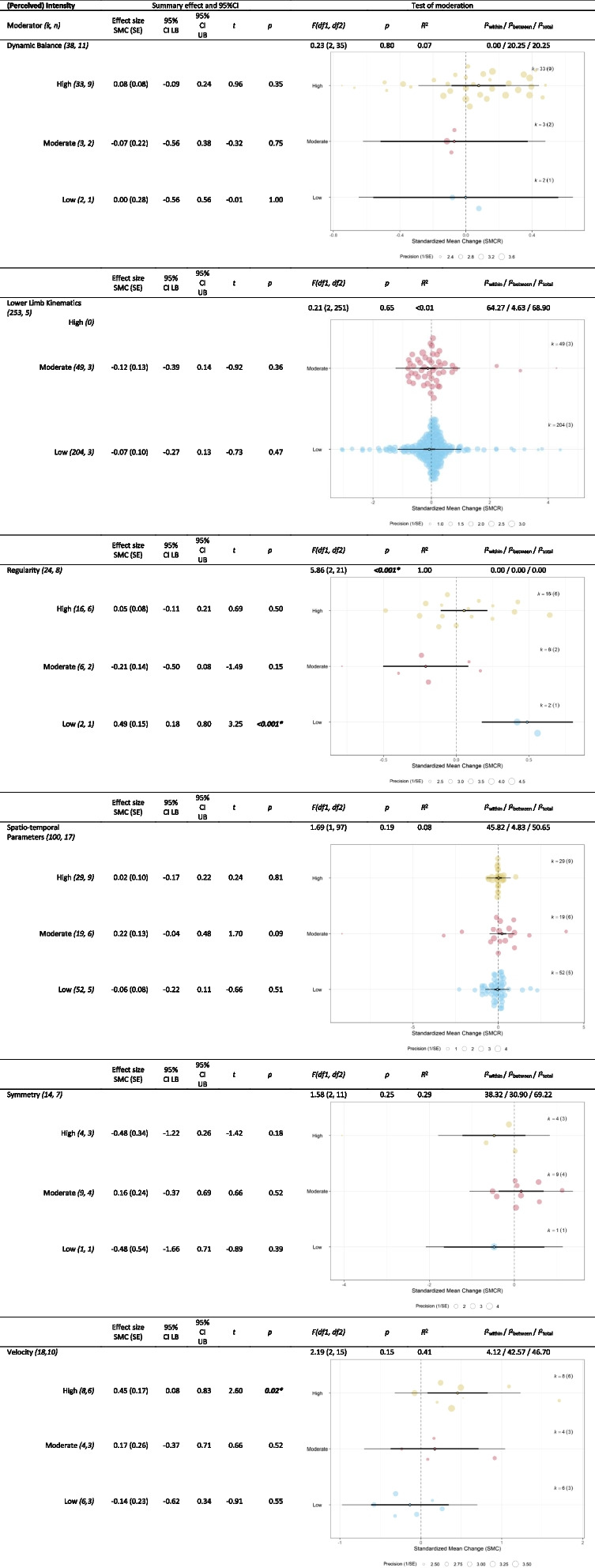
Outcomes of moderator analyses of ‘(perceived) intensity’ on different clusters of gait parameters (*r* = 0.25)

* Statistically significant results, also in italic and bold. *CI* confidence interval, *df* degrees of freedom, *k* effect sizes, *LB* lower bound, *n* datasets, *PI* prediction interval, *SMCR* Standardized mean change, *SE* standard error, *UB* upper bound. In the orchard plots the estimate (open circle), the confidence interval (horizontal thick black line), and the prediction interval (horizontal thin line) can be distinguished. Furthermore, the size of the color filled circles corresponds with the precision of the effect size. Note that in the Lower Limb Kinematics cluster the High condition is missing since none of the studies that looked at lower limb kinematic gait parameters used a high intensity protocol

## Sensitivity analyses and analysis of bias

### 3.5.1 P-curve analysis

**Fig. 6:**
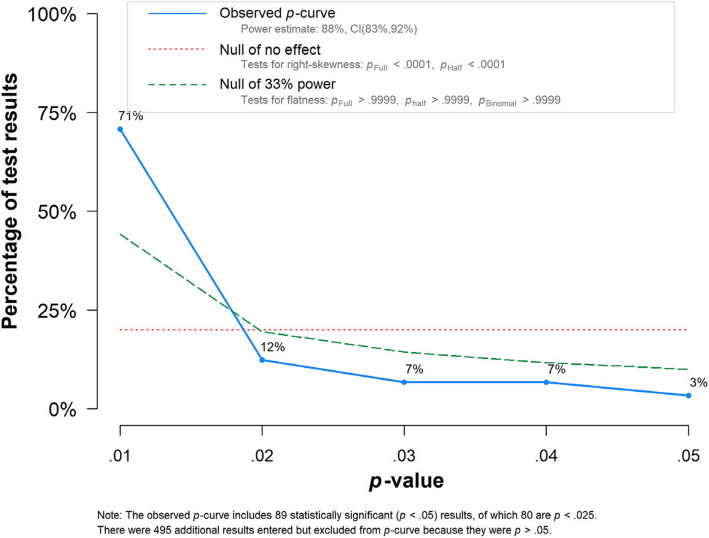
P-curve analysis on all (absolute) effect sizes (*r* = 0.25)

The *p*-curve analysis shows that most of the effect sizes were non-significant, and the analysis shows an ability to detect true effects with the power estimation: 88%, 95% CI [83%, 92%] (Fig. 6).

### 3.5.2 Sensitivity analysis and publication bias

The (contour-enhanced) funnel plots of all the clusters appeared considerably symmetrical. Meta-analysis on bias-corrected pooled point estimates, showed findings similar to our analysis of the clusters. When excluding possible outliers, this again showed similar results to our cluster analysis. Analysis on selective reporting showed no signs of *p*-hacking. Overall, we did not find major indications of bias due to selective reporting or publication bias that changed the interpretation of our results. All the sensitivity analyses are presented in the Supplementary Materials (8. Outcomes meta-analysis – Sensitivity analysis).

### 3.5.3 Post factor power calculation

The post factor power calculation showed that with a mean effect size of SMCR = –0.016 the minimum number of studies needed ranged between 655 to 8053. Simulation input ranged from τ = 0.10, ω = 0.00, *ρ* = 0.20 to *τ* = 0.40, ω = 0.30, *ρ* = 0.70, where tau (τ) reflected heterogeneity, omega (ω) reflected within-study effect size range, and rho (ρ) indicated the correlation between effect size within studies. See Supplementary Materials (6. Power analysis Post factor R-script) for more details.

## Discussion

In this study, we aimed to understand whether and how exercise-induced fatigue affects gait in community-dwelling older adults, and what specific elements of fatiguing protocols moderate the effects. Our meta-analyses indicated that exercise-induced fatigue in general affects gait parameters. However, these general effects could not be specified within any of the six clusters of the gait parameters that we identified (i.e., Dynamic Balance, Lower Limb Kinematics, Regularity, Spatio-temporal Parameters, Symmetry, and Velocity) as effects were not one-directional and hence non-distinguishable from zero. Furthermore, the (perceived) intensity, type of activity or duration of fatiguing exercises appeared not to be significant moderators of the general effects.

## Overall effects were not reflected in clusters of gait parameters

As hypothesized, gait parameters change as a result of fatiguing activity. This is shown in the significant small to moderate changes on absolute data and is in line with existing literature. Nonetheless, analysis of absolute data makes it difficult to understand in what direction gait parameters are affected. Due to the large amount, and variety of gait parameters derived from all included studies in our meta-analyses, clustered gait parameters were needed to analyze the non-absolute data. Ideally, similar gait parameters would have been clustered, but the limited number of similar gait parameters found in the included studies made this not possible. As a result, different gait parameters were combined into clusters. Within the clusters, no significant changes in gait parameters were observed. These findings could potentially be explained by several factors. First, within clusters, there are conflicting or opposing parameters. We deemed that a pooled zero-effect as a result of (multiple) conflicting or opposing mechanisms may only appear in the Spatio-temporal Parameters cluster, where cadence was combined with single support time, stance time, step time, and swing time. So, in the case fatigue increases cadence, typically (one of) the five other spatio-temporal parameters will decrease. In the other clusters, less or no opposing effects of pooled parameters were expected. Second, between studies varying or opposite effects were reported. As shown in Table 3, only a minority of gait parameters had the same direction (all increase or decrease) post-fatiguing. The identified differences could be explained by the diverse ways to measure gait parameters. For example, in the analysis data from studies that used a treadmill was combined with studies that used overground walking to measure gait parameters. Although gait parameters on treadmill and overground are comparable, they are not always similar [90], and this combined analysis may have affected our findings. Third, besides conflicting parameters within clusters and between studies, an explanation could be the between-subject variation. The close-to-zero effects could also indicate that the pre- to post-fatiguing effects vary within studies. As shown in the summary of findings table the majority of findings are non-significant (Table 1). This could be explained by the possibility that the effects of fatigue on gait parameters, or the compensatory mechanisms that people show when fatigued [91], are not uniform. For example, a change in gait velocity could be a way to counter the feeling of instability [92]. In contrast to the review of Santos et al. [6], which showed an increase in gait velocity post fatigue, we found no significant changes in walking speed. Santos et al. [6] found this increase in speed with the four papers they included and mentioned that it could possibly be the result of a warm-up effect. In our meta-analysis, we used 26 effect sizes from 15 datasets and only 65% of the effect sizes indicated an increase in walking speed. This finding is in line with other research, indicating that participants potentially make changes in walking speed or other gait parameters to cope with (feelings of) fatigue or instability [93].

## Potential moderators did not explain the (absence of) fatigue effects on clusters of gait parameters

In the moderator analysis of activity type, we distinguished walking activity from other activities. This was based on the assumption that walking is a rhythmic activity that affects multiple joints and muscles per cycle and therefore the fatiguing effect on gait may differ substantially from (isokinetic) single joint activities (i.e. knee flexion / extension). Moreover, walking activities are part of everyday life and relate close to the gait measures that we focused on in our study. Nonetheless, the type of activity appeared not to moderate the effects of fatigue in any of the clusters. Apparently, all types of activity (i.e., walking, and non-walking) have an effect on gait performance.

Furthermore, both (perceived) intensity and duration showed not to moderate the effects of fatiguing exercise on gait parameters. With respect to task dependent fatigue, taking only one moderator into account might not be sufficient to limit the variation in underlying mechanisms and sites associated with fatigue [24]. In daily living, activities show an interplay between the type of activity, the (perceived) intensity, and the duration of exposure, depending on the task requirements. Ideally, an interaction effect of moderators could give more insight into this ecological interplay and thereby possibly identify specific elements of fatiguing protocols. Unfortunately, due to the insufficient number of effect sizes, it was not possible to look at interaction effects between moderators in our meta-analysis.

## Methodological aspects and limitations

The use of the algorithm-assisted ASReview resulted in a broader search, which probably resulted in including more papers than with a more conventional search strategy. In ASReview Lab, only 10% of the papers we found in our search were screened. Although a substantial number of papers was not screened by the authors, we are confident that all relevant papers are included. This latter was also underlined by simulations done in ASReview Lab. Furthermore, based on the algorithm, finding more relevant papers would not even out to the time spent [31]. In hindsight, the search could have been broadened even more. We now used the “NOT” operator to filter out based on title, but we could have trusted the software to filter for us. The use of the "NOT" operator to filter based on titles may have excluded studies where control group data could have been utilized. Nonetheless, including all these studies would possibly make the learning phase more complex, which could result in more studies that had to be assessed for full text but did not meet our inclusion criteria. Fortunately, contact with authors led to the inclusion of a control group from an article that had been filtered out based on the “NOT” operator. However, the extent of potentially missed control groups remains unclear. To analyze all the gait parameters in clusters with sufficient effect sizes, the clusters as introduced by Lindemann [37] were used. Although the work of Lindemann and other classifications is primarily focused on spatio-temporal gait parameters [37, 39, 94], it gave us tools to cluster all our gait parameters in six groups only. Although we tried to be as consistent as possible with the original clustering, we did include gait parameters that have not been clustered in the Lindemann’s framework before. Our clustering of the gait parameters thereby had a broader spectrum of the gait parameters per cluster than originally reported by Lindemann [37]. Another result of this clustering is that we could not compare our findings with literature on individual gait parameters other than velocity.

When comparing our a priori power analysis and post factor power analysis, two quite different numbers of studies that needed to be included for sufficient power were found. This is explained by two factors. First, prior to the start of this meta-analysis, there was no multilevel power calculation known by the authors. Second, in the a priori power calculation, an estimated effect size was used that was higher than the effect size that came out of the analysis. Therefore, it could be possible that our zero effect is a result of too little effect sizes that are included in our study.

An aspect that might have had an impact on our outcomes is the fact that we included eight articles (based on seven data sets) that reported on prolonged activity instead of a pre- vs. post-fatiguing exercise set up. In the studies with prolonged activity, fatigue was assumed as a consequence of activity and took data from the first minutes as pre-fatiguing measurement and from the last minutes as post-fatiguing measurement. As shown, gait speed is faster at the beginning and end of 400-m walk test and significantly lower in the middle, possibly because older adults are motivated to finish [95, 96]. The post-outcome measures might overestimate actual gait parameters, resulting in smaller differences between pre- and post-assessments.

An aspect influencing the calculated effect sizes, and consequently the pooled effect, was the need to impute correlations. This approach assigned the same correlation value across all pre- and post-measurements. While using actual correlations instead of imputed values would likely yield results closer to the true effects, the authors believe that such adjustments would not significantly alter our findings. The presence of heterogeneity influenced the precision of the true effect estimation. Our meta-analysis indicates that such variability in underlying studies (in terms of fatiguing protocols and outcome measures) limits the robustness of the findings. Thus, without more strict experimental control and without reducing the methodological differences across studies, the presence of heterogeneity would still be a serious problem for future meta-analytical attempts. Therefore, we feel that the GRADE certainty rating, which was (very) low in most cases, is a good reflection of the available papers in the field. Studies not only varied in the types of fatiguing exercises employed, but also in the methods used to measure the outcomes. As mentioned before, treadmill walking or overground walking could have different effects on gait parameters [90], but also the different overground walking distances could have had impact on gait parameters [97, 98].

## Implications and recommendations

We now know that exercise-induced fatigue changes gait parameters in general (first hypothesis), but we could not pin-point these changes to specific directions, nor within clusters of gait parameters. Furthermore, no evidence was found to support our hypotheses that fatiguing exercises with a longer duration (second hypothesis), or a higher (perceived) intensity (third hypothesis), or that consisted of walking activities (fourth hypothesis) would show greater changes in gait parameters. This could be the result of the possible different strategies to cope with fatigue between participants, but is also a result of too many methodological inconsistencies in primary studies. Researchers in the field of gait analysis should report more similar gait parameters and measure these outcomes in more similar ways [98–100]. Similar recommendations for standardization of fatiguing protocols have been emphasized in reviews on the effects of exercise-induced fatigue addressing other target populations [101, 102]. More standardization in different aspects would make future systematic reviews and meta-analysis easier to conduct and could make such analysis more conclusive than the current work. Nonetheless, from current work we can conclude that it is important that older adults are aware of their changed physical (walking) abilities after fatiguing exercise in order to protect themselves from possible adverse effects. Although duration, type of activity and (perceived) intensity did not significantly moderate our findings, these moderators are building blocks when pre-scribing exercise [103]. Therefore, both researchers and therapists should know that regardless of the fatiguing exercise, older adults will show changes in walking performance. More practical, it seems that researchers can choose a fatiguing protocol that best suits their question, is possible in their lab settings, matches with their participants preferences or is the least invasive for participants, as long as they manage to fatigue participants. Therapists should be aware that the walking performance of older adults may be reduced or (un) intentionally improved, in relation to fall prevention, when fatigued. Thereby, underlining the need for tailor-made fall prevention, and taking sufficient rest after fatiguing exercises.

Research focusing on the effects of exercise-induced fatigue and gait parameters, should try to explain why exercise-induced fatigue could lead to non-uniform changes between participants. Do older adults actively choose a different movement behavior when fatigued? And do they make changes in gait parameters because they are aware of adverse fatiguing effects, are topics that are not well researched yet.

## Conclusion

In walking, exercise-induced fatigue leads to small to moderate changes in gait parameters. These changes cannot be attributed to specific clusters of gait parameters. Furthermore, we could not identify specific elements of fatiguing exercise that lead to changes in gait parameters, as the type of activity, duration, or (perceived) intensity of the exercise did not moderate our findings. This may have a three-fold explanation: (1) older adults respond in both positive and negative ways to fatiguing exercise in their performance, (2) the type of gait parameters and how they are measured is too heterogeneous, or that (3) different moderators or interactions between moderators are necessary to explain these pooled close-to-zero effect sizes.

## Data Availability

The datasets generated and/or analyzed during the current study are available in the Figshare repository, 10.21943/auas.25651671 and 10.21943/auas.25652523. The following materials can be found on our Figshare pages: 1. Search and inclusion related documents. 2. R-script and excel file for the interrater reliability. 3. Risk of Bias and GRADE documents. 4. Summary of Findings table. 5. Data file for the main analysis (coding form). 6. Analysis files (main, sensitivity, and power analysis). 7. Outcomes of the analyses with the different between measurement correlations. 8. Outcomes of the sensitivity analysis. 9. PRISMA checklist Abstract + Main.

## Abbreviations

DB: Dynamic balance
CI: Confidence interval
CINAHL: Cumulative index to nursing and allied health literature
df: Degrees of freedom
ES: Effect size
k: The number of effect sizes
LB: Lower bound
LLK: Lower limb kinematics
MeSH: Medical subject headings
n: The number of datasets
NI: No information
PI: Prediction interval
PRISMA: Preferred reporting items for systematic reviews and meta-analyses
PROSPERO: International prospective register of systematic reviews
Reg: Regularity
REML: Restricted maximum-likelihood estimator
SE: Standard error
SMCR: Standardized mean change using raw score standardization
ST: Spatio-temporal parameters
Sym: Symmetry
UB: Upper bound
Vel: Velocity
